# COGNITIVE RESERVE IN AGING AND PRECLINICAL ALZHEIMER’S DISEASE

**DOI:** 10.1093/geroni/igae098.1680

**Published:** 2024-12-31

**Authors:** Anja Soldan, Corinne Pettigrew, Marilyn Albert

**Affiliations:** Johns Hopkins University, Baltimore, Maryland, United States; Johns Hopkins University School of Medicine, Baltimore, Maryland, United States; Johns Hopkins University School of Medicine, Baltimore, Maryland, United States

## Abstract

There is large inter-individual variability in cognitive and brain aging trajectories, which have been related to differences in cognitive reserve (CR), brain mainenance, and the influence of genetic and environmental factors across the lifespan. This presentation will discuss recent evidence regarding CR using data from the BIOCARD study, which includes ~ 300 individuals who were cognitively unimpaired and primarily middle-aged at baseline (mean baseline age=57) and have been followed with clinical and cognitive evaluations, brain imaging, and blood and cerebrospinal fluid (CSF) collection for over 20 years. This work has shown that higher levels of CR, indexed using years of education, literacy, and vocabulary measures, are associated with higher baseline cognitive performance and a delayed onset of Mild Cognitive Impairment, even in the presence of Alzheimer’s disease (AD) pathology and neurodegenration. By comparison, CR measures were not related to levels or rates of change in AD biomarkers (e.g, CSF and blood levels amyloid and tau), suggesting that CR influences cognitive and clinical outcomes through mechanisms other than AD pathology accumulation. Instead, further results suggest that measures of CR are associated with white matter hyperintensity burden and white matter microstructural integrity. Additionally, other modifiable lifestyle factors known to influence cognitive outcomes (e.g., physical activity, sleep quality and quantity, and engagement in stimulating leisure activities) are associated with measures of resting-state functional brain connectivity. Taken together, these findings suggest that modifiable lifestyle factors may promote cognitive reserve and resilience by improving aspects of functional and structural brain connectivity and cerebrovascular health.

